# Composition of Coffee Beans Influenced by Bioprocessing with Selected Bacteria

**DOI:** 10.3390/foods14071143

**Published:** 2025-03-25

**Authors:** Paulina Pakosz, Anna Bzducha-Wróbel, Beata Drużyńska, Ewa Majewska, Rafał Wołosiak

**Affiliations:** 1 Division of Food Quality Assessment, Department of Food Technology and Assessment, Institute of Food Sciences, Warsaw University of Life Sciences (WULS-SGGW), 159 Nowoursynowska Street, 02-776 Warsaw, Poland; beata_druzynska@sggw.edu.pl (B.D.); ewa_majewska1@sggw.edu.pl (E.M.); rafal_wolosiak@sggw.edu.pl (R.W.); 2 Department of Biotechnology and Food Microbiology, Institute of Food Sciences, Warsaw University of Life Sciences (WULS-SGGW), 159C Nowoursynowska Street, 02-776 Warsaw, Poland; anna_bzducha_wrobel@sggw.edu.pl

**Keywords:** coffee beans, green coffee modification, coffee composition, roasting, bioactive compounds, luwak coffee, kopi luwak

## Abstract

Coffee quality can be modified with microorganism addition during post-harvest processing. While most studies focus on yeasts and lactic acid bacteria, other species identified in the digestive tract of palm civets might also contribute to the quality of luwak coffee. Bacteria akin to those identified in palm civets’ gastrointestinal tract or feces were evaluated for their potential to modify coffee bean composition. Among those, *Bacillus subtilis* ATCC 6633, *Gluconobacter* sp. KKP 3751 and *Lactiplantibacillus plantarum* ATCC 4008 exhibited strong growth in green coffee extract. The use of these bacteria significantly changed the amounts of basic coffee components (taste and aroma precursors), and slightly altered bioactive compound levels in green and roasted beans. The influence of fermentation duration was evaluated using *L. plantarum*. A stationary growth phase and positive changes regarding phenolic content were achieved after 24 h of fermentation. Overall, the use of bacteria can influence bean composition, offering the potential to create unique coffee products.

## 1. Introduction

The characteristics of coffee are significantly influenced by factors related to its production, from geographical origin and growing conditions, ending at roasting and brewing methods [1,2]. Post-harvest processing, one of the initial stages in coffee processing, primarily aims to separate coffee beans from cherries and reduce their moisture content (≤10–12%), minimizing the risk of mold growth and ensuring safety. This stage is crucial for the formation of precursors to the flavor and aroma compounds created during roasting, resulting in coffee with diverse sensory quality [3,4,5,6]. 

Coffee beans are typically separated from their fruits using either the dry or wet method. The dry processing method (also known as the natural method) involves drying of the entire coffee fruit (from 15 days up to 6 weeks) and then removing dehydrated husk (pulp, mucilage, parchment). In contrast, the wet processing method involves pulping of the coffee cherries, fermentation of beans still covered with mucilage (12–72 h), and subsequently washing, drying and hulling. The semi-washed method involves the same beginning stages as the wet processing; however, after fermentation beans are partially dried (moisture ~35%), then undergo husk removal and are further dried to obtain the required final moisture content. The honey process is yet another modification of the wet method, in which coffee beans are dried with the mucilage still attached; the hulling process is carried out as the final step [1,5,7,8,9]. There is also unique coffee processing that requires animal participation, such as in the production of luwak coffee (kopi luwak). Predominantly produced in Indonesia, this process involves palm civets (*Paradoxorus hermaphroditus*), which are known for the consumption of ripe coffee fruits. The green beans are collected from the civets’ feces and further processed [10]. Other examples of animal-processed coffee are ivory coffee and jacu coffee [11,12].

The influence of microorganisms on coffee composition and quality has been extensively studied. Experiments introducing starter cultures containing fungi and/or bacteria in various kinds of post-harvest processing methods resulted in reduction in processing time and products with distinctively altered characteristics [3,13,14,15]. New processing methods, such as anaerobic fermentation, carbonic maceration or digestion methods, have been developed to meet the growing demand for unique coffee products [5,11,16]. These processes were also applied to the green coffee beans to improve the quality of more widely available coffee material compared with fruits [17,18,19,20]. 

Luwak coffee, an example of digestion processing, gained significant attention, particularly concerning the microorganisms present in the digestive tract or feces of the palm civet, i.e., their types and role in the creation of the coffee’s quality. Muzaifa et al. [21] reported that the number of lactic acid bacteria (LAB) and non-LAB in a civet’s digestive tract is similar, at approximately 7–9 logarithm of colony forming units/mL (logCFU/mL); however, non-LAB showed slightly higher values in all examined parts of the digestion tract. The research of Suhandono et al. [22] focused on the identification of culturable bacteria present in three parts of the digestive tract and reported the habitation of bacterial genus such as *Bacillus*, *Pseudomonas*, *Pantoea*, *Escherichia*, *Lactobacillus*, *Ochrobactrum* and *Kocuria*. Later research showed that bacteria from the *Gluconobacter* genus are also common in the digestive tract of palm civets and their metabolism might contribute to the quality of kopi luwak [23]. Knowledge about microorganisms occupying the digestive tract of palm civets creates the opportunity to produce luwak-like coffee without the participation of the animal itself. It could increase production rates without the need for the captivity of civets and caged production of this unique coffee [10]. Having in mind the possibility that the selection of the sweetest and ripest coffee fruits might be of higher importance than the digestion process itself, such knowledge also allows for the further verification of microorganisms’ importance in kopi luwak production.

Regarding bacteria as starter cultures, both post-harvest modifications and luwak coffee research have been focused on LAB and their role in coffee fermentation, which might be connected to their general safety, common usage in food manufacturing, as well as natural presence during post-harvest processing and high enzymatic activity. Bacteria such as *Lactobacillus plantarum*, *Lactococcus lactis* subs. cremoris and *Lactobacillus brevis* were identified during coffee processing and were further examined in research [4,15,19,22,24,25,26]. To date, no coffee fermentation with *Gluconobacter* has been performed and bacteria from *Bacillus* genus (e.g., *B. subtilis*, *B. cereus*) has only been attempted few times, resulting in coffee quality improvement [27,28,29]. Given that research on the digestive tract of civets has revealed a diverse range of bacterial genera beyond LAB, it is appropriate to consider and analyze their potential to modify coffee through fermentation.

The objective of our study was to evaluate the possibility to use selected bacteria from the species identified in the digestive tract of palm civet in green coffee bean modification. The study consisted of three experiments; each experiment resulted in the selection of bacteria for further steps. Basically, a range of bacteria were evaluated for their ability to grow in a coffee environment (Experiment 1), and three chosen cultures of different genera were checked for their ability to change the composition of green and roasted coffee (Experiment 2). The character of the changes was further investigated in regards to various fermentation times (Experiment 3) and optimal modification time was proposed.

## 2. Materials and Methods

### 2.1. Materials

The chemicals used in this study were purchased from SigmaAldrich (Buchs St. Gallen, Switzerland), Chempur (Piekary Śląskie, Poland), Alchem Group (Toruń, Poland), Avantor Performance Materials Poland S.A. (Gliwice, Poland) and VWR International Sp. z o.o. (Gdańsk, Poland). Basic chemicals used in coffee extract preparation and spectrophotometric analyses were acquired from Chempur (sodium carbonate, sodium hydroxide, copper(II) sulfate pentahydrate, sodium potassium tartrate tetrahydrate, Folin–Ciocalteu reagent), VWR International Sp. z o.o. (acetone) and SigmaAldrich (sodium dodecyle sulphate). The latter one also provided compounds that were used as standards across all analyses (sucrose, fructose, bovine serum albumin, gallic acid, 3-caffeoylquinic acid [3-CQA]). The caffeine standard was obtained from Alchem Group, while the glucose standard was purchased from Chempur. SigmaAldrich provided acetonitrile and Avantor Performance Materials Poland S.A. supplied methanol and acetic acid solution, which were used as mobile phases in instrumental analyses. For the cultivation of the selected bacterial strains, tryptic soy broth (TSB) and de Man, Rogosa and Sharpe medium (MRS) were purchased from BTL sp. z o.o. (Łódź, Poland) and Millipore, Merck (Darmstadt, Germany), respectively.

Green *Coffea arabica* (further referenced as Arabica) and *C. canephora* var. robusta (further referenced as Robusta) beans were purchased from local roastery LaCava sp. z o.o., originating from Rwanda, and processed via the wet method. A day before modification, 75 g of green beans were combined with 100 mL of distilled water in a glass bottle and sterilized (121 °C, 0.1 mbar, 20 min) in order to ensure a lack of other microorganisms during further processing. 

Bacterial strains (*B. subtilis* ATCC 6633, *Gluconobacter* sp. KKP 3751, *Lactiplantibacillus plantarum* ATCC 4008, *Levilactobacillus brevis* DSMZ 20053 and *L. brevis* from Biolacta, Poland) collected in the Museum of Pure Cultures at Department of Food Biotechnology and Microbiology, Faculty of Food Sciences, Warsaw University of Life Sciences WULS-SGGW were applied for experiments. Cultivations of the tested bacteria were carried out at 30 °C for 24 h in the respective media: TSB for *B. subtilis*, glucose and yeast extract medium (GY; 50 g glucose, 10 g yeast extract, 1000 mL distilled water) for *Gluconobacter* sp. and MRS for LAB. To prepare the inoculum, cells were separated from the cultivation medium by centrifugation (6000 rpm, 4 min) and dispersed in sterile water to obtain a standardized cell concentration at the level 0.5 in McFarland’s scale, which corresponds to approximately 10^8^ CFU/mL.

The aforementioned growth media were later used in the preparation of control samples during screening and in plate counting, with the addition of agar (2%).

### 2.2. Experiment Design

Analyses were divided into three parts. Experiment 1 entailed the screening of bacteria to evaluate their ability to grow in the green coffee extract. Strains which showed the greatest ability to grow in coffee extract were selected to use in Experiment 2, which consisted of green coffee bean fermentation. Lastly, Experiment 3 focused on conducting fermentations with varying durations, utilizing a single bacterial culture.

#### 2.2.1. Experiment 1—Screening of Bacterial Strains’ Growth

As an experimental medium, the water extract from Arabica beans created during green coffee beans sterilization (as described in Section 2.1) was used. This extract was evaluated for the content of soluble peptides, carbohydrates, caffeine and 3-CQA. The methods further described in Section 2.4 were utilized; all analyses were performed in quadruplicates.

The growth of the examined bacteria was monitored using a microplate reader (Multiscan Sky, Thermo Fisher Scientific, Waltham, MA, USA). Within the 96-well microplate, 150 μL of sterile green coffee extract, or sterile medium in the case of controls (as described in Section 2.1), was inoculated with 10 μL of bacterial inoculum, previously diluted tenfold (10^5^ CFU). Each combination of bacterium and medium was prepared in quadruplicate. The experiment was conducted at 30 °C for 48 h with no shaking. This temperature was selected as the most suitable for all selected bacterial species. Measurements of optical density (OD, 600 nm), which corresponds with the cell concentration, were performed at 0, 24 and 48 h.

#### 2.2.2. Experiments 2 and 3—Fermentation Procedure and Analysis

Sterile green coffee beans (prepared as described in Section 2.1) were combined with 20 mL of inoculum (10^8^ CFU/mL) and the overall volume was adjusted to 200 mL with sterile distilled water. Composition of fermentative liquids from Arabica and Robusta bean modification was analyzed at the beginning of the process; the methods described further (in Section 2.4) were utilized. The fermentation conditions included a temperature of 30 °C, agitation at 125 rpm, durations of 24 h for Experiment 2, and 6, 12, 24 and 48 h for Experiment 3. The control samples were prepared in the same manner, substituting bacterial inoculum with an equivalent volume of sterile water. Two independent samples were prepared for each coffee and bacterium (Experiment 2), as well as coffee and duration (Experiment 3) combinations.

Fermentation was monitored by determination of the pH changes and enumeration of bacterial cells via plate method; both analyses were performed at the initiation and completion of the fermentation. pH values were measured with a pH meter (pH 50+ DHS, XS instruments) which was calibrated before analysis using buffer solutions (pH = 4.0 and 7.0). The measurement was performed four times for each sample. Plate counting required a serial, tenfold dilution of fermentative liquid to calculate the number of studied bacteria. The plates were incubated at 30 °C for 24 h, with the exception of *Gluconobacter* sp. which required 48 h of incubation. The colonies were enumerated and the number of bacterial cells was calculated. Analyses were performed in duplicate, inoculating samples from three consecutive decimal dilutions.

### 2.3. Coffee Preparation

After fermentation, green coffee beans were rinsed with distilled water (200 mL) and dried in an air oven at 40 °C to ensure their preservation for further analysis. Samples of green coffee (25 g) were roasted using iKAWA Pro V2 sample roaster (max temperature 213 °C; total time 5.75 min). Beans from both processing steps (green and roasted) were ground using a laboratory grinder (MF 10 basic, IKA, Warsaw, Poland) running at 3500 rpm and equipped with a 2 mm sieve. Ground coffee was used to prepare extracts with a methanol and sodium dodecyl sulfate solution (1%) using previously published methodology [30]. The finalized extracts were kept in the freezer (−20 °C) pending analysis.

### 2.4. Coffee Chemical Analysis

The amount of dry matter in green and roasted coffee beans was determined by measuring the weight loss after drying (104 °C) 2 g of ground coffee to a stable mass. The obtained values were used in further calculations. The color of ground coffee material in the CIELab system was measured using Chroma Meter CR-400 (Konica Minolta Co., Ltd., Tokyo, Japan) which was calibrated with a white standard before analysis. Measurements were repeated four times for each analyzed sample.

Both green and roasted coffee underwent analysis considering fundamental constituents (sugars, soluble peptides) which are integral to overall coffee quality as well as bioactive constituents, such as caffeine and polyphenols. 

Coffee carbohydrates (glucose, fructose, sucrose) were analyzed by high-pressure liquid chromatography (HPLC) with an evaporative light-scattering detector (ELSD). The analyzed compounds were separated on the Supelcoil LC-NH2 column (Sigma-Aldrich Co. LLC, Burlington, MA, USA, 25 cm × 4.6 mm, 5 μm), and a mixture of acetonitrile and water (78:22) was used as a mobile phase (isocratic flow, 1 mL/min). Then, 3-CQA and caffeine contents were also analyzed using HPLC, but with a photodiode detector (PDA). The Kinetex 5u C18 100A column (Phenomenex, Torrance, CA, USA, 150 × 4.6 mm) was used for separation, and 0.1% acetic acid in water with methanol mixed in 87:13 ratio was used as the mobile phase (isocratic flow, 1 mL/min). In both analyses, columns’ temperature was set to 30 °C and the injection volumes were in the range of 5–25 μL. Compounds were identified by comparison to the results obtained for standard solutions analyzed under the same conditions.

Soluble peptide content in coffee was quantified using the Lowry method [31], while total polyphenols content was assessed with the Folin–Ciocalteu method [32]. As standards, bovine serum albumin and gallic acid solutions in proper solvents were used, respectively. Measurements were performed using a spectrophotometer (UVmini 1240, Shimadzu, Kyoto, Japan) with the following wavelengths: 750 nm in the case of Lowry method and 765 nm for Folin–Ciocalteu method. Detailed descriptions of all methods used in this study are given in our previous article [30].

### 2.5. Statistical Analysis

Statistical analysis was used in order to evaluate the significance of the differences observed between samples in each experiment. Suppositions regarding the normal distribution of values and equality of model’s residuals were analyzed using Shapiro–Wilk and Levene tests, respectively. For small datasets, assumptions were assessed visually through graphical methods using Q-Q plots and residual plots. Furthermore, a one-way analysis of variance (ANOVA) or ANOVA with Welch correction was used, depending on the fulfillment of aforementioned assumptions. If the significance of the analyzed factor was proofed, the mean values were compared in order to create homogenous groups using Tukey’s HSD test or Wilcoxon test with Bonferroni correction. In cases where only two sets of results were compared, t-Student’s test was also used. Principal component analysis (PCA) and cluster analysis were performed to further compare the green and roasted beans obtained in Experiments 2 and 3. In the case of the latter, Euclidean distance and Ward’s method were used for the distance metric and clustering algorithm, respectively. The samples were compared using all data obtained in the course of the experiments, with the exception of simple sugars’ (glucose, fructose) concentrations. ANOVA analyses were performed in R Statistical Software v4.3.2 [33] with confidence level set at 0.05, and PCA and cluster analysis were performed using STATISTICA 13.3 software (StatSoft, Inc., Tulsa, OK, USA).

## 3. Results and Discussion

### 3.1. Experiment 1: Screening of Bacterial Strains’ Growth

As the first experiment, bacteria from species comparable to those inhabiting the gastrointestinal tract of palm civets were subjected to growth screening which was performed using a plate reader. Arabica coffee extract created during the sterilization of beans was used as an experimental medium; the composition of this extract was as follows: soluble peptides 97.33 ± 0.73 g/L, sucrose 40.60 ± 1.23 g/L, 3-CQA 10.72 ± 0.10 g/L and caffeine 3.40 ± 0.03 g/L. Simple sugars (glucose, fructose) were present in trace amounts.

**Figure 1:**
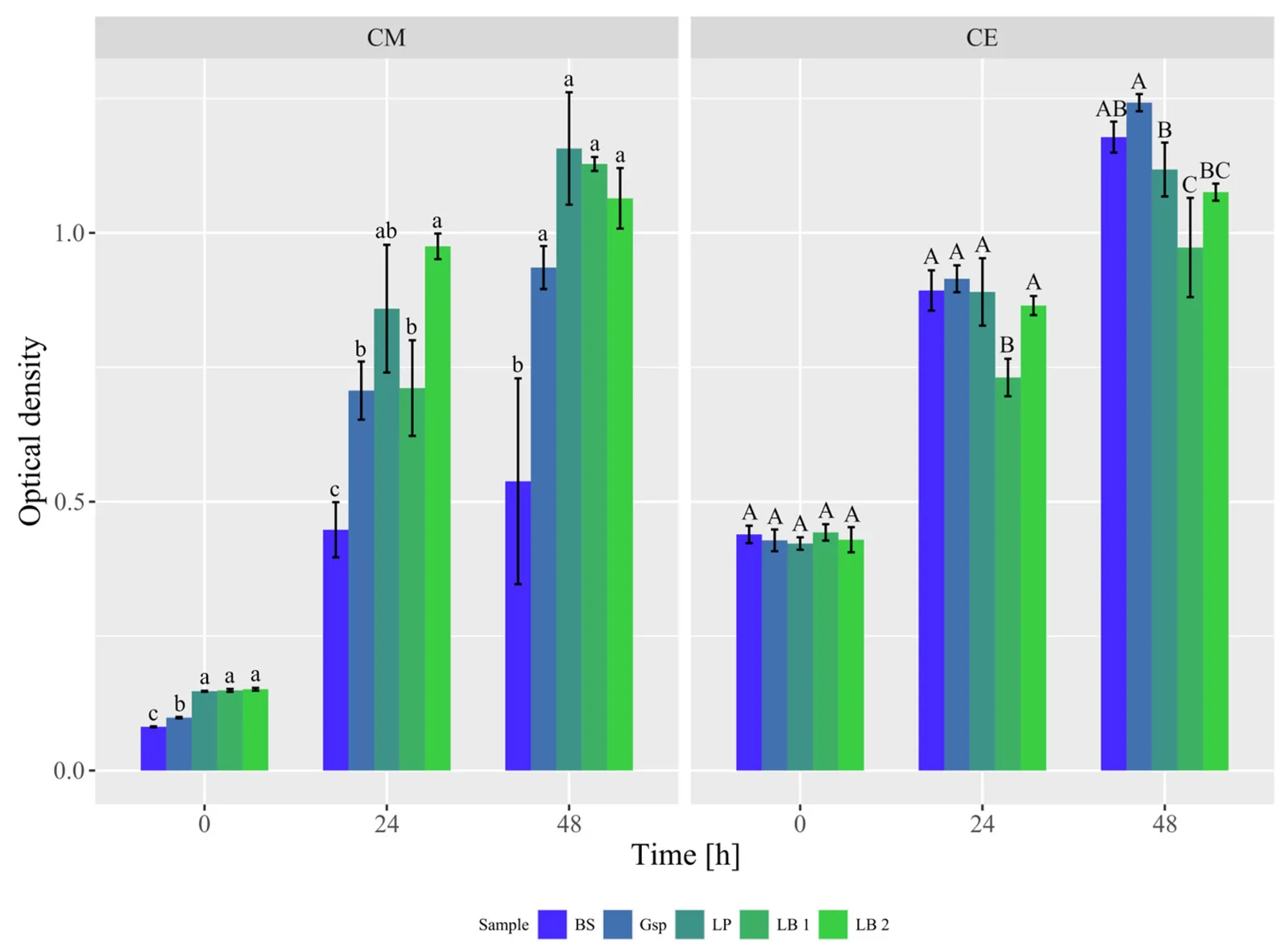
Optical density (600 nm) of control media and coffee extract inoculated with selected bacteria measured during growth ability screening (Experiment 1). CM—control medium (tryptic soy broth, glucose and yeast extract medium, de Man, Rogosa and Sharpe medium for *B. subtilis*, *Gluconobacter* sp. and LAB, respectively); CE—green coffee extract. BS—*B. subtilis* ATCC 6633, Gsp—*Gluconobacter* sp. KKP 3751, LP—*L. plantarum* ATCC 4008, LB 1—*L. brevis* DSMZ 20053, LB 2—*L. brevis* from Biolacta, Poland. For each time period, letters indicate the homogenous groups created after the analysis of variance; lowercase letters signify differences between controls, and uppercase letters between fermented samples.

The ODs of the samples made with coffee extracts and their respective controls (with appropriate synthetic grow media) are presented in Figure 1. The experimental medium—green coffee extract designated in Figure 1 as CE—had a higher OD compared to the control media by approximately 0.3, which can be explained by differences in their compositions. After 24 and 48 h, an increase in OD values can be seen in both coffee extracts and controls. Among the samples prepared with coffee extract, inoculation with *L. brevis* DSMZ 20053 resulted in the lowest OD, which corresponds to its low growth capability.

Since the media used in the experiment exhibited different ODs from the beginning, OD changes after each incubation period for all prepared samples and controls were calculated (Table S1 in the Supplementary Materials). In the case of *B. subtilis*, observed increases in OD in coffee extracts were greater than those observed in the TSB medium (by 123.8% and 161.8% after 24 and 48 h, respectively). During the first incubation period, the growth of *Gluconobacter* sp. in coffee extract was approximately 80% of the change observed in the reference medium (GY); however, after 48 h, the observed values were similar (97%). The lowest relative changes in coffee extracts (51–71% in relation to changes in MRS medium) were observed in the case of examined LAB (*L. plantarum*, *L. brevis* DSMZ 20053, *L. brevis* from Biolacta, Poland). For each LAB, extending incubation resulted in a decrease in observed differences between samples with the coffee medium and their respective controls, which was particularly evident in *L. brevis* produced by Biolacta, Poland.

These findings underscore that coffee constituents can sustain the growth of the selected microorganisms to varying degrees. Throughout the assessed duration, LAB exhibited an overall lower ability to grow in the tested coffee medium compared to *B. subtilis* and *Gluconobacter* sp. Even though LAB are known to metabolize phenolic acids, it is possible that their high concentration in the experimental medium—more than 10 g/L of 3-CQA—could negatively affect the growth rate of the examined bacteria [34]. The results obtained for strains of *L. brevis* highlight the variability in growth capabilities within the same bacterial species. While *B. subtilis* and *Gluconobacter* sp. are aerobic bacteria, LAB are aerotolerant anaerobes, which might also suggest that their growth might have been restrained by the presence of oxygen [23,35,36].

Bacteria demonstrating the most pronounced growth ability in the green coffee extract were selected for the second experiment. Based on the statistical analysis of both OD values and calculated OD changes, *B. subtilis* ATCC 6633 and *Gluconobacter* sp. KKP 3751 were chosen. Among LAB commonly utilized in research, *L. plantarum* ATCC 4008 was designated as it exhibited strong and the most uniform growth in this group during both examined time intervals. It is possible that the characteristics of these bacteria’s growth and their metabolism could have potential advantages for the coffee modification process, such as expediting the achievement of bacterial growth’s stationary phase and overall shortening of fermentation time, as well as a greater potential for the modification of green coffee beans themselves.

### 3.2. Experiment 2: Coffee Fermentation with Selected Bacterial Strains

The bacteria selected in Experiment 1 were used in the fermentations of green Arabica and Robusta coffee beans, each lasting 24 h. The composition of the liquid medium in which the modification of coffee beans was carried out was analyzed at the beginning of the experiment. The modification process was characterized by pH measurements and enumeration of bacteria cells at the beginning and at the end of fermentation time.

The medium obtained from Arabica beans exhibited a higher concentration of sucrose than extracts from Robusta beans (16.28 ± 0.65 mg/mL and 10.02 ± 0.25 mg/mL, respectively); in both cases, glucose and fructose were present in trace amounts. Robusta liquid medium was characterized with greater amounts of soluble peptides (38.91 ± 2.56 mg/mL), 3-CQA (4.10 ± 0.22 mg/mL) and caffeine (3.94 ± 0.31 mg/mL) compared to Arabica extract (22.67 ± 1.34 mg/mL, 3.73 ± 0.08 mg/mL, 1.90 ± 0.14 mg/mL, respectively). Such differences were expected due to the different composition of beans and are in accordance with the literature [37].

The initial cell count in the coffee mixtures was close to 6 logCFU/mL, except for *Gluconobacter* sp., where that number was closer to 7 logCFU/mL. After 24 h of fermentation, all examined bacteria demonstrated growth in the experimental environment proved by an increase in cell numbers, reaching 7.9–8.8 logCFU/mL (Table 1). Ngamnok et al. [19] isolated *L. plantarum* and *Paenibacillus motobuensis* from the civet intestines and utilized them in green coffee beans fermentation based on their enzymatic activity. In their study, *L. plantarum* reached peak growth after 12 h of fermentation—from 9 to 12 logCFU/mL—after which a constant cell concentration was achieved. Wang et al. [20] used another representative of LAB, *L. lactis* subsp. *cremoris*, to enhance coffee flavor. They noted no significant difference regarding cell concentration after 24 h of fermentation with and without glucose supplementation; however, supplemented fermentation exhibited greater growth after 12 h, which was followed by a decrease in cell count. Similar observations for coffee fermentation with *L. plantarum* were also reported by Kim et al. [17], showing a 1.5 logCFU/mL increase in cell count after one day of fermentation; in comparison with other LAB used in their study, *L. plantarum* exhibited the lowest cell concentration at this time. Our results, considering the growth of LAB representative during coffee fermentation, align with existing literature. To the best of our knowledge, there are no data regarding the growth of *B. subtilis* and *Gluconobacter* sp. during coffee fermentation using either coffee fruits or green beans. Based on cell count after 24 h obtained in this experiment, the growth ratios for *B. subtilis* and *L. plantarum* were similar (approximately 140% of the onset counts), while *Gluconobacter* sp. exhibited a lower value (approximately 120%). All selected bacteria showed greater growth capability than in Experiment 1. In this experiment, fermentative liquid was diluted, which resulted in a lower concentration of phenolic compounds, which could have favored bacterial growth. Moreover, continuous extraction processes from coffee beans might have supported the availability of compounds necessary for bacterial growth during 24 h of modification. Such processes might have been further affected by interactions between microorganisms and the coffee matrix.

**Figure 2:**
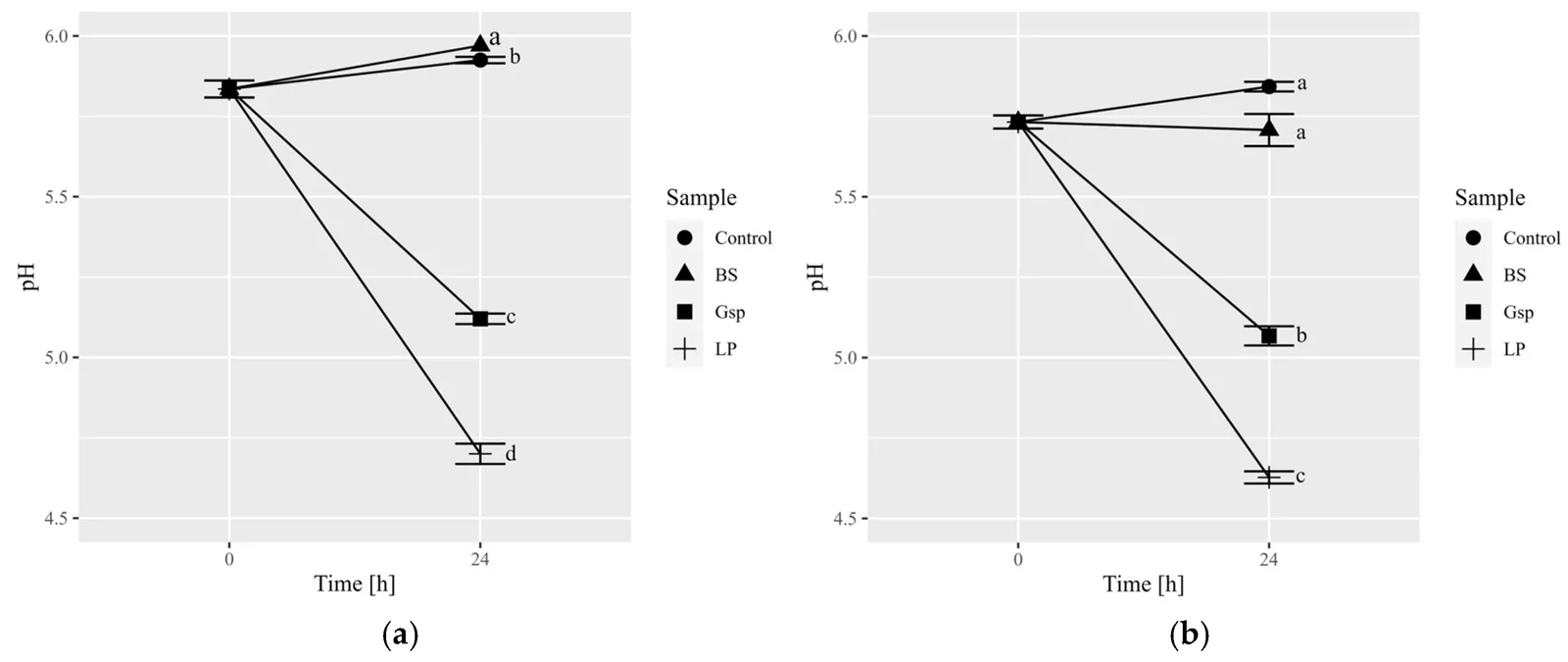
pH values of Arabica (**a**) and Robusta (**b**) fermentative mixtures prepared during Experiment 2. Control—non-fermented, coffee beans soaked in water, BS—*B. subtilis* ATCC 6633, Gsp—*Gluconobacter* sp. KKP 3751, LP—*L. plantarum* ATCC 4008. For values obtained after 24 h of fermentation, lowercase letters indicate homogenous groups created after the analysis of variance.

The acidity values (pH) for Arabica and Robusta fermentative mixtures at the onset of fermentations were 5.84 and 5.73, respectively (Figure 2). Upon the completion of fermentation, values for controls in both coffee varieties increased by 0.1 (Figure 2), which could be attributed to green coffee metabolism in wet conditions [19] and continuous extraction. Fermentation with *B. subtilis* also elevated pH values, which resulted in production of mixture with the highest pH value among all examined samples (6.03 for fermented Arabica). Muzaifa et al. [28] observed a decrease in pH values after 48 h of coffee fermentation using *B. subtilis* isolated from civet’s gastrointestinal tract, although under different conditions compared to study presented in this paper (i.e., use of depulped coffee beans, lack of sterilization of the coffee material). Moreover, *B. subtilis* strains exhibited various enzymatic activities, which indicates possible differences in their metabolism—e.g., *B. subtilis* used in soybeans fermentation resulted in an increase in pH value during this process [38]—and may elucidate the observed discrepancies. Conversely, fermentations with *Gluconobacter* sp. and L. plantarum led to decreased pH values (Figure 2), attributable to their carbohydrate-metabolism-yielding acids, mainly acetic acid and lactic acid, respectively [39]. The results for *L. plantarum* are in accordance with the previously published work regarding the usage of LAB in such a process [9,19,20]. Notably, samples fermented with *L. plantarum* consistently exhibited the lowest pH value among all tested variants in both Arabica and Robusta, potentially linked to the production levels and inherent acid strength of lactic acid compared to acetic acid. Due to its high pH-lowering capability, other strains of *L. plantarum* have also been utilized in on-farm experiments to facilitate the fermentation process [15,40].

#### 3.2.1. The Influence of Bacterial Strains on Green Coffee Color and Composition 

Following bean preparation (drying and grinding), the color attributes of fermented and control green coffee beans were analyzed in the CIELab system and comprised of lightness (L*), redness (+a*) and yellowness (+b*). In general, Arabica beans exhibited higher color parameter values compared to Robusta beans, which can be seen in Figure S1(1–3) in the Supplementary Materials. The L* and b* values for controls and samples fermented by *B. subtilis* were similar, while fermentation by acid-producing bacteria (*Gluconobacter* sp. and *L. plantarum*) resulted in varied, significant increases of those values. A similar trend was observed for a* values of Arabica beans, however no statistically significant differences were found between them. In the case of Robusta beans, processing with *Gluconobacter* sp. resulted in significant increase of beans’ redness in comparison with other fermented beans; all fermented samples did not differ significantly from control.

Carbohydrates and proteins are the main components of green coffee beans. These compounds undergo various changes during roasting (i.e., Maillard reaction, degradation, caramelization), thus playing a significant role in creation of coffee taste and aroma [6,37]. Furthermore, carbohydrates and proteins are primary nutrients for bacteria during fermentation, which suggests that additional fermentation might have an effect on their concentration in beans.

The raw coffee material contained low amounts of simple sugars not detected in Arabica; in Robusta beans, the amounts of fructose and glucose were 0.05 and 0.06 g/100 g dry matter (dm), respectively. Sucrose content in untreated Arabica and Robusta beans was 4.13 and 2.80 g/100 g dm, respectively. Concentration of simple sugars and sucrose in green coffee beans obtained in Experiment 2 are presented in Table 2. In controls, the amount of sucrose was lower than those found in the raw green coffee beans indicative of continuous extraction taking place during the preparation and processing of coffee material. The differences in the concentrations of simple sugars might have resulted from structural changes in coffee during sample preparation, allowing for their determination.

The fructose concentration in beans fermented by *B. subtilis* was similar to concentration in control samples. These beans also demonstrated the highest glucose content (0.22 and 0.19 g/100 g dm for *B. subtilis*-fermented Arabica and Robusta beans, respectively) among the samples. Conversely, *Gluconobacter* sp.-fermented green beans exhibited the opposite trend: those samples contained the highest fructose content (0.25 and 0.21 g/100 g dm for fermented Arabica and Robusta beans, respectively) and moderate glucose levels, all significantly higher than the amounts found in the controls. Fermentation with *L. plantarum* led to the most significant decrease in fructose and glucose concentrations. Sucrose concentration in coffee beans decreased after fermentations with *B. subtilis* and *Gluconobacter* sp. Among fermented Robusta samples, beans fermented with *L. plantarum* exhibited the greatest amount of sucrose (2.44 g/100 g dm); however, it did not differ significantly from the amount found in *B. subtilis*-fermented beans. In the case of Arabica fermented samples, *B. subtilis*-fermented beans were shown to have the highest concentration of this sugar (3.09 g/100 g dm), which, again, was not significantly different from the value found in *L. plantarum*-fermented sample (2.89 g/100 g dm).

*B. subtilis* utilizes various carbohydrates, including those analyzed in this study [41]. Bacteria from the *Gluconobacter* genus thrive on glucose, and only some species are capable of using fructose and sucrose in this manner. Nonetheless, most acetic acid bacteria are capable of utilizing sucrose for the production of acids [39,42]. All those literature data support the results reported here regarding *Gluconobacter* sp.-fermented coffee.

The effect of coffee modification with LAB on carbohydrate concentrations has been previously studied and similar changes were reported. As part of their experiment, Therdtatha et al. [26] conducted the fermentation of Arabica green beans using *L. plantarum,* reporting no changes in sucrose concentration post process. During a field experiment, coffee processed by the wet method was inoculated with the selected *L. plantarum* strain. After 24 h of fermentation, a significant decrease in reducing sugars content in fermentation mass was observed, which was explained by conversion to organic acids; overall faster acidification of inoculated fermentation mixture was observed compared to spontaneous fermentation [15]. During fermentation with other LAB representatives, researchers reported a significant decrease in glucose and fructose levels, with sucrose concentration either remaining unchanged or slightly increased due to a higher rate of extraction compared to its metabolism. Additionally, supplementation with glucose resulted in a greater production of lactic acid compared with the non-supplemented sample [17,20]. These results indicate that sucrose may not be an optimal carbon source for LAB during coffee fermentation. 

Summing up, the changes in sugar concentrations could be attributed to bacterial metabolism and progressive extraction from coffee beans. Furthermore, microbial enzymes could interact with the complex structure of coffee material resulting in the release, utilization and/or degradation of other carbohydrates. It should also be noted that the enzymatic activity of some bacteria could result in the breakdown of sucrose into simple sugars. This could provide an explanation for higher concentrations of glucose in *B. subtilis*-fermented beans and fructose in *Gluconobacter* sp.-fermented ones compared to control beans: the extraction process might have been altered overtime due to a change in simple sugars concentrations in the fermentative mixtures.

Generally, the soluble peptide content in Arabica beans was lower than in Robusta ones (9.9–11.2 and 14.7–17.6 g/100 g dm, respectively). Fermented Arabica beans exhibited no significant differences in peptide concentration. In the case of Robusta, *B. subtilis*-fermented beans differed significantly from *Gluconobacter* sp.- and *L. plantarum*-fermented samples; the latter ones exhibited lower peptide concentrations. All fermented Robusta samples did not differ significantly from control (Table 2). 

Similarly to carbohydrates, lower amounts of peptides in the controls compared to raw coffee material (20.32 and 23.58 g albumin/100 g dm in green Arabica and Robusta, respectively) indicate their extraction during sample preparation, as observed by other researchers [20]. A decrease in protein concentration in green beans was also observed by Wibowo et al. [29], who employed a bacteria consortium isolated from the saliva of *Arctictis binturong* Raffles, 1821 in coffee fermentation. The initially observed downtrend was succeeded (after 8 h) by a rise in soluble protein concentration, possibly linked to the modification of coffee structural proteins into smaller, more soluble and more biologically active peptides [43]. A decrease in protein concentration might also be connected to their breakdown into amino acids and further bacterial metabolism; such observations were previously reported for various coffee fermentations [20,26]. It is worth noting that such declines in protein and/or amino acid concentrations were not universally observed. Ngamnok et al. [19] reported a lack of protease activity during the first 24 h of fermentation process suggesting either their production during stationary growth phase of bacteria or no need for their production as greater amounts of carbohydrates were available for enzymatic actions.

Finally, the analyses focused on the compound characteristics of coffee. Among these, caffeine is the best known, primarily responsible for coffee’s stimulatory effects and also contribution to bitterness. Additionally, coffee is a rich source of antioxidants, predominantly phenolic compounds. Chlorogenic acids are the most important phenolics of coffee, among which caffeoylquinic acid isomers are the most abundant [7,9,12,44]. Untreated green Arabica and Robusta beans contained 0.61 and 1.06 g/100 g dm caffeine, 1.52 and 1.98 g/100 g dm total polyphenols as well as 1.91 and 1.85 g/100 g dm 3-CQA, respectively.

All prepared samples exhibited a lower concentration of bioactive compounds than raw coffee material, which, once again, could be associated with extraction during preparation and processing of coffee beans. All Robusta samples exhibited greater amounts of caffeine compared to Arabica beans, which is explained by inherent differences between coffee species (Table 2). Specifically, caffeine levels ranged between 0.63 and 0.66, and 1.03 and 1.13 g/100 g dm for Arabica and Robusta beans, respectively. In general, fermentation with selected bacteria resulted in slight, mostly insignificant changes of the amount of caffeine in green coffee samples; only in the case of Robusta beans fermented with *L. plantarum*, a significant increase was noted. Total polyphenols and 3-CQA contents exhibited consistent trends considering fermentation or its absence (Table 2). Generally, bioprocessing did not significantly affect the levels of fundamental antioxidants present in green coffee; fermentation with *L. plantarum* resulted in both an increase and decrease in the analyzed parameters in Arabica and Robusta beans, respectively.

The literature reports suggest that additional green coffee processing with bacteria might lead to varying degrees of caffeine concentration reduction, which might be due to extraction or microbial enzyme activity [19,26,29]. On the other hand, Therdtatha et al. [26] reported an increase in the concentration of this alkaloid in Robusta coffee beans fermented by yeast cocktail and *L. plantarum*, while Kim et al. [17] suggested that caffeine levels might be influenced in various ways depending on the starter culture used. Data regarding total polyphenols and 3-CQA contents paint a similarly ambiguous picture. Evidence exists for increased total polyphenol, chlorogenic acid and other phenolic acid (i.e., caffeic and quinic acids) contents [17,19]. Moreover, Wang et al. [20] reported a rise in quinic acid concentration in green coffee beans fermented by *L. lactis* subsp. *cremoris,* accompanied by a decrease in chlorogenic and caffeic acid levels, while fermentations conducted by Therdtatha et al. [26] suggested that LAB alone do not influence these parameters significantly. The results presented in this paper mostly align with the notion of no significant effect of additional fermentation on the content of analyzed bioactive compounds in green coffee, consistent with the aforementioned literature. Enzymes produced by microorganisms could release phenolic compounds from complex coffee structures. The loss of phenolics observed here could be attributed to facilitated extraction during modification, as well as their further metabolism by bacteria. Additionally, the hydrolysis of 3-CQA to simpler phenolic acids may account for its loss [17,19]. It was also proposed that phenolic acids could be degraded to volatile compounds during fermentation [20]. Interestingly, fermentation with *L. plantarum* had different effects on Arabica and Robusta coffee beans, which suggests that material specificity might be important for the course of additional bioprocessing by microorganisms.

**Figure 3:**
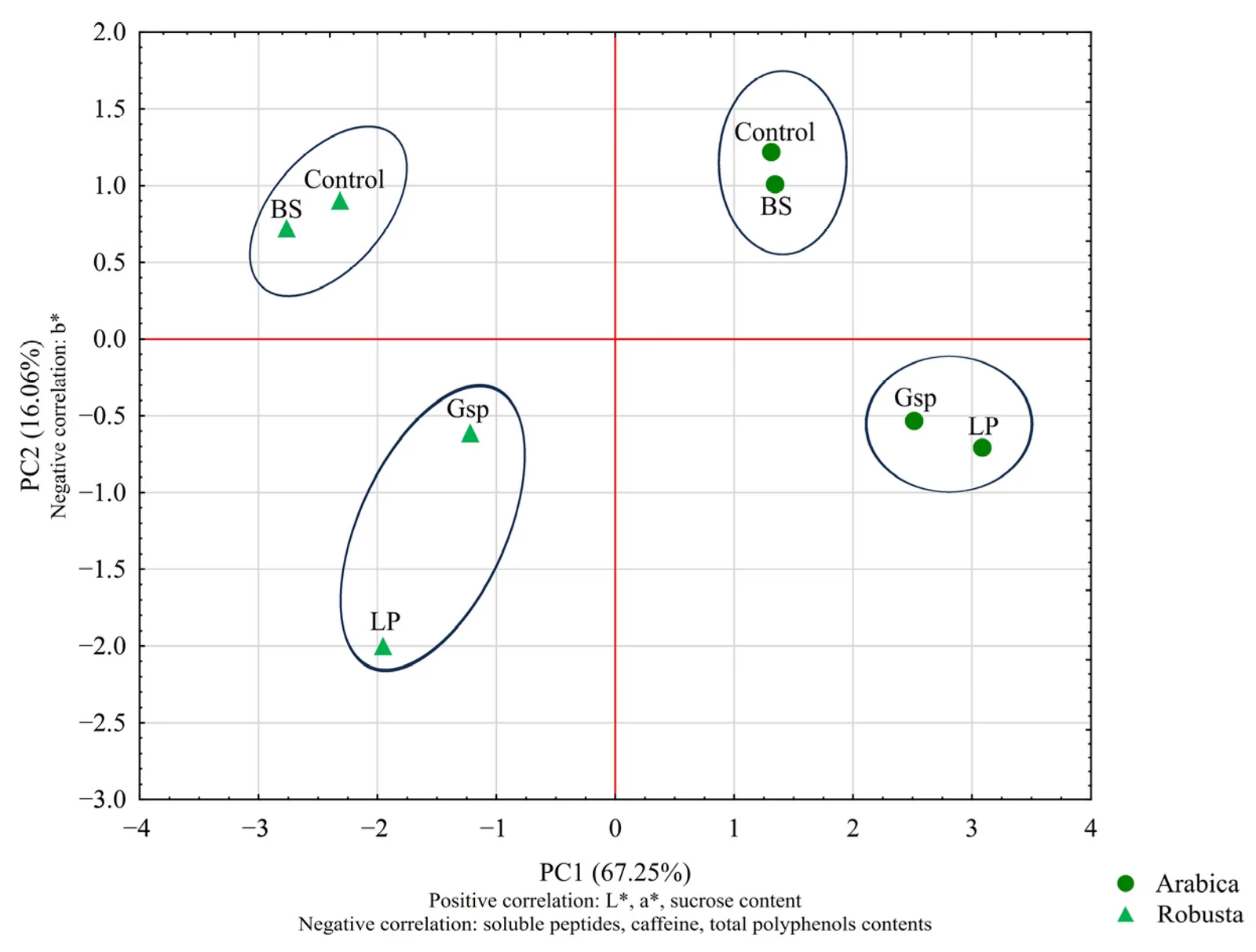
Principal component analysis (PCA) score plot derived from color and composition data for green coffee beans fermented with different bacterial strains and their control samples (Experiment 2). The samples were divided into clusters (illustrated by the circles) based on cluster analysis. The samples are described in the same manner as in previous figures.

Figure 3 shows the results of PCA and cluster analysis for green beans obtained in this experiment. Correlations of experimental parameters indicate their increase or decrease (for positive or negative correlation, respectively) with the increase in principal component value. The 3-CQA content was not strongly correlated with either of the principal components, which shows that its changes were not significant enough to differentiate coffee samples. After analysis, principal components 1 and 2 (PC1 and PC2, respectively) explained more than 83% of variance observed between samples. Based on PC1, clear distinction between all Arabica and Robusta samples can be seen. Overall, the differentiation of samples presented in Figure 3 aligned with the discussion presented above. The effect of fermentation with *Gluconobacter* sp. and *L. plantarum* distinctly differentiated both coffee beans from their controls and *B. subtilis*-fermented samples, which was further underlined by cluster analysis. 

#### 3.2.2. The Influence of Roasting on Fermented Beans’ Color and Composition

The fermented coffee beans and controls underwent roasting under identical conditions and were analyzed in the same manner as the green ones. All changes in the analyzed coffee attributes and constituents—such as reduction in all color parameters as well as carbohydrates, soluble peptides, caffeine, total polyphenols and 3-CQA concentrations (Table 2)—align with findings reported in the literature [6,44,45]. They stem from reactions like caramelization, degradation or Maillard reactions occurring during the heat treatment of green coffee, which is when compounds responsible for coffee color, taste and aroma are generated. As those changes are well-described in the literature, the following section will focus mainly on changes in roasted coffee, which could be associated with additional fermentation.

The color of roasted beans of Arabica and Robusta was similar (Figure S1(4–6) in Supplementary Materials). As in the case of green beans, the color of roasted coffee was most significantly affected by acid-producing bacteria (*Gluconobacter* sp. and *L. plantarum*), resulting in significantly lower color values compared to other samples. This observation is consistent with the literature. It was suggested that a higher content of acids in green beans accelerates caramelization during roasting, which not only results in darker coloration, but also a more intense caramel aroma in the final products [17,20]. In roasted beans, the amount of soluble peptides exhibited the same trend as in the case of green ones; specifically, all samples prepared from Robusta beans were characterized by a higher content of soluble peptides in comparison to those prepared from Arabica beans. Additional fermentation resulted in the increase in soluble peptide content in roasted beans. In the case of Arabica, only fermentation with *L. plantarum* resulted in significant change from the control sample; fermented Robusta beans did not differ significantly from control sample. Following roasting, significant variations in the concentrations of bioactive compounds were observed mainly in Robusta beans. In comparison to the roasted control beans, fermented Robusta samples were characterized by higher levels of caffeine as well as significantly elevated 3-CQA concentration in *B. subtilis*- and *L. plantarum*-fermented samples. In the case of Arabica, an increase in total polyphenol content was also observed after fermentation with *B. subtilis* and *L. plantarum*, but not significantly different from the control; regarding the 3-CQA content, an insignificant decreasing trend was observed.

**Figure 4:**
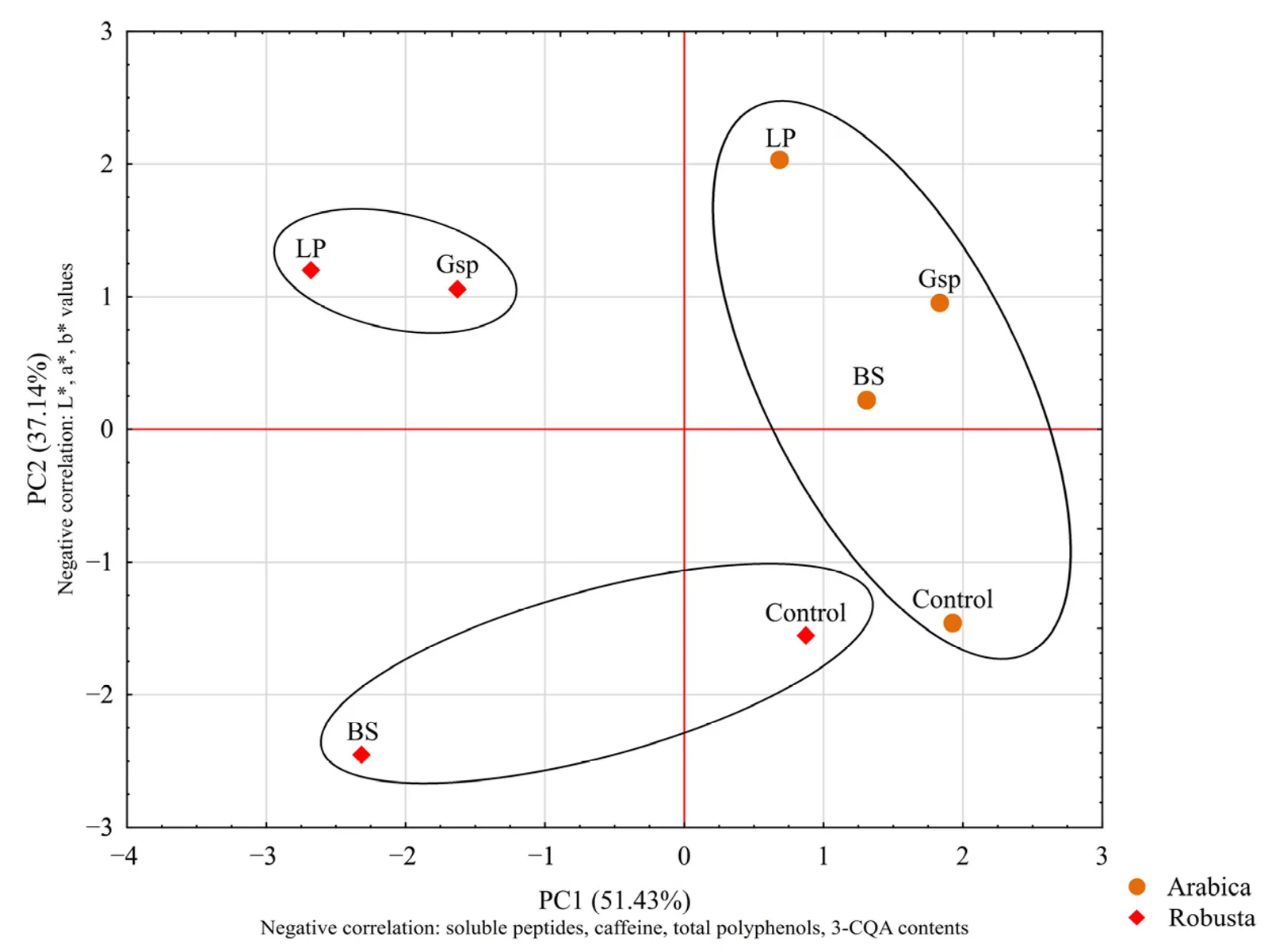
PCA score plot derived from color and composition data for roasted coffee beans fermented with different bacterial strains and their control samples (Experiment 2). Samples were divided into clusters (illustrated by the circles) based on cluster analysis. Samples described in the same manner as in previous figures.

The PCA and cluster analysis for roasted coffee beans is presented in Figure 4. In the case of Arabica beans, changes in color values (correlated with PC2) were more pronounced and resulted in clear distinction between fermented and control samples. In the cluster analysis, those samples were grouped together, suggesting their general similarity. For Robusta beans, more pronounced changes regarding both color and composition were observed, especially after fermentation with *Gluconobacter* sp. and *L. plantarum*.

These results indicate that, apart from extraction, additional processing with selected bacteria influenced the composition of coffee beans; the effect was more pronounced in the case of green beans. It can be speculated that such changes might result in altered roasted coffee quality, which shows the potential of such modification (in combination with appropriate roasting level and brewing method) to produce coffee products with unique quality. Furthermore, the obtained results showed a possibility to retain or increase the amount of primary antioxidants in both green and roasted coffee, which might have a positive effect on human health.

### 3.3. Experiment 3: Influence of Time on Coffee Fermentation with the Selected Bacterium

The duration of the fermentation process is a crucial factor influencing the composition of coffee. In Experiment 3, we tested the influence of time of fermentation (6, 12, 24 and 48 h) with the selected bacterium on the composition of both Arabica and Robusta beans; growth dynamics and changes in pH value of fermentative mixtures were also analyzed. Based on the previous results, *L. plantarum* ATCC 4008 was selected for this experiment due to its potential for enhancing bioactive compound levels in coffee beans. Fermented and control samples were prepared and analyzed following the methodology used in Experiment 2.

**Figure 5:**
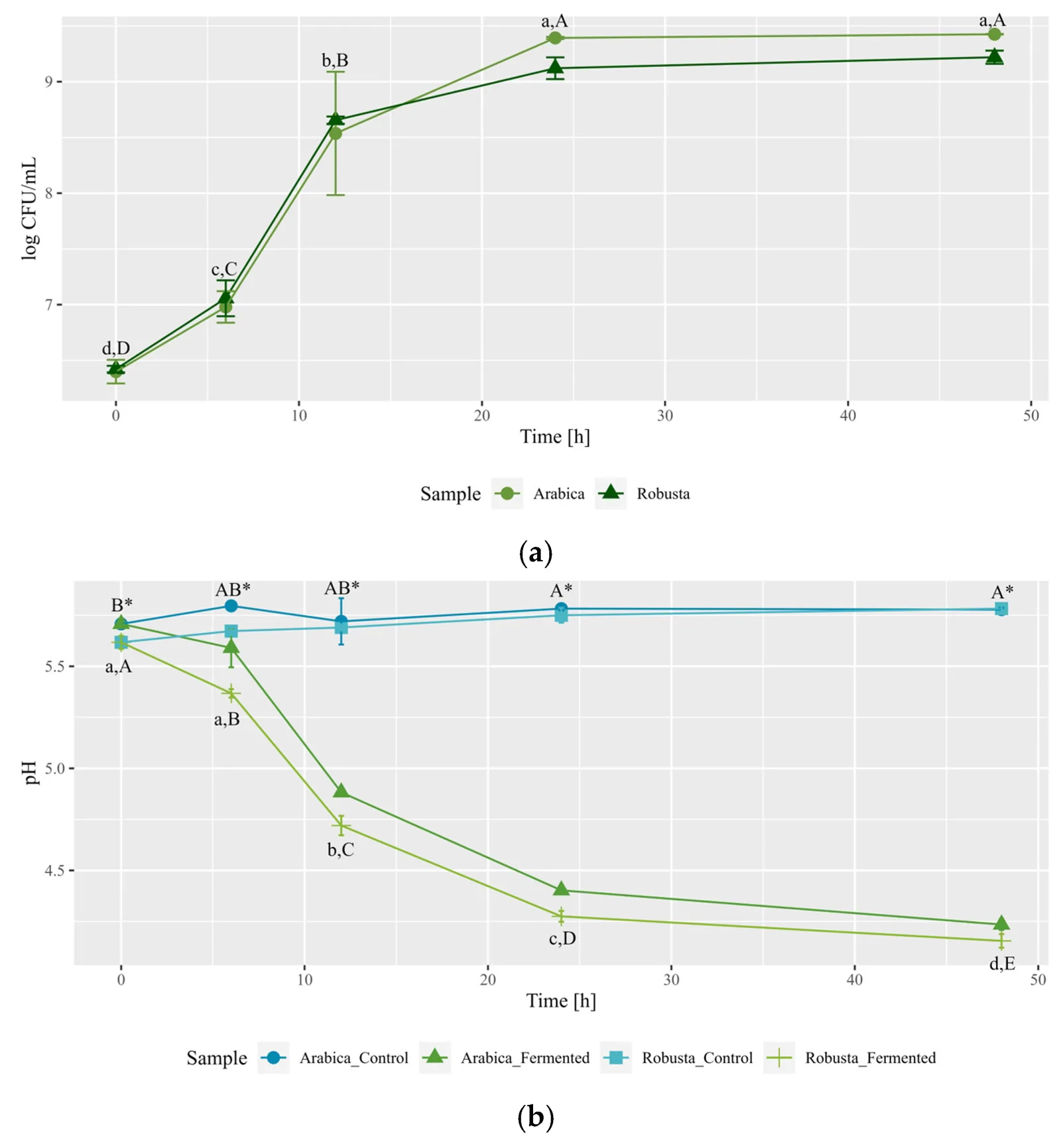
Cell count of *L. plantarum* ATCC 4008 (**a**) and pH values (**b**) of Arabica and Robusta samples fermented with varying durations obtained in Experiment 3. Letters indicate homogenous groups created after the analysis of variance; lowercase letters signify differences between Arabica samples, uppercase letters between Robusta samples and * signifies differences between respective coffee species in controls. Results without letter indicators did not differ significantly.

The starter cell counts were around 6 logCFU/mL. The greatest increase in the bacterial population was observed between 6 and 12 h of fermentation, resulting in a rise by 1.56 and 1.59 logCFU/mL for Arabica and Robusta beans, respectively (Figure 5a). Subsequently, a slight increase was observed and a relatively stable number of bacteria was reached, indicating a stationary growth phase after 24 h. Although the fermentation patterns of Arabica and Robusta beans were similar, Arabica exhibited slightly higher bacterial concentrations after 24 and 48 h of fermentation. The stationary phase of growth generally begins when readily available nutrients in the medium are exhausted. At this point, bacterial cells transition from a growth-oriented state to one focused on survival, which requires various morphological and physiological adaptations, such changes might alter metabolites produced during this phase, potentially affecting the quality of fermented product [46,47,48].

The acidity mirrored the observed growth patterns (Figure 5b). Prolonged fermentation led to a more pronounced decrease in pH values. A difference between 6 and 12 h of fermentation was the most prominent; for Arabica and Robusta mixtures, the decrease in pH was 0.71 and 0.65, respectively. Similar changes were observed by Pereira et al. [15], where in *L. plantarum*-inoculated fermentation, the greatest decrease in pH value was observed after 12 h. Consistent with the bacterial growth dynamics, further declines in pH values were observed with extended durations; the magnitude of those changes progressively weakened.

#### 3.3.1. The Influence of Fermentation Duration on the Color and Composition of Green and Roasted Coffee Beans

In Supplementary Materials, Figures S2(1–3) and S3(1–3) show the color of green fermented and control samples prepared with various fermentation times. In controls, lightness and yellowness slightly increased, peaking after 12 or 24 h of process before declining. Simultaneously, redness intensified with enhanced modification. Fermented samples exhibited a rise in all color parameters for both Arabica and Robusta beans. Interestingly, in both coffee species, the results obtained after 6 and 12 h of fermentation were significantly lower than those obtained after longer durations (24 and 48 h). 

Upon roasting, Arabica controls generally showed an increasing trend in redness and yellowness with longer processing times; in Robusta beans significant increase was observed at the earlier stages, however with prolonged duration, values of color parameters decreased to the levels observed after 6 h of fermentation. All fermented beans exhibited a significant decreasing pattern, attributed to heightened acidity and increased caramelization during roasting, as noted previously [17,20]. Identical to the color changes observed in green beans, values obtained after shorter fermentation durations (6 and 12 h) significantly diverged from those obtained after 24 and 48 h of processing (Figures S2(4–6) and S3(4–6)).

The results pertaining to carbohydrate levels and soluble peptide concentrations in green and roasted coffee beans are presented in Table 3. The amounts of simple sugars (fructose and glucose) in fermented beans exhibited a decreasing trend, reaching trace levels or becoming undetectable after 12 h for Arabica beans and after 24 h for Robusta beans. De Carvalho Neto et al. [40] observed total consumption of glucose after 12 h and a significant decrease in fructose after 14 h of *L. plantarum*-inoculated fermentation of Arabica depulped beans. The controls showed a similar, but not significant decreasing trend. Sucrose concentration decreased in both fermented samples and control beans. Soluble peptide content demonstrated a more pronounced decrease in controls. In fermented green Arabica beans, relatively consistent values were observed across all fermentation durations, while in Robusta beans, more pronounced changes were noted. 

Roasting generally decreased the amount of fundamental compounds in all analyzed samples. The controls attained the highest peptide content after 12 and 24 h for Arabica and Robusta, respectively. A wide range of values obtained for fermented and roasted coffee beans made it difficult to detect significant trends related to the duration of the process. On average, modified Arabica beans contained generally higher amounts of soluble peptides compared to their respective controls after appropriate modification time, while Robusta beans showed generally lower average values.

Investigation into the effect of the biomodification time on the contents of bioactive compounds in coffee beans (caffeine, polyphenols, 3-CQA) resulted in rather interesting observations. The controls of both coffee species showed a significant downward trend in caffeine content, which was common for green and roasted beans (Table 3). Fermentation with *L. plantarum* resulted in the stabilization of caffeine content during processing, which in turn resulted in higher caffeine levels in coffee beans fermented for longer durations (24 and 48 h) compared to their respective controls; this trend was again consistent across pre- and post-roasting stages. Similar tendencies can be seen with regard to the total polyphenols content (Table 3): considering only fermented samples, significantly higher values were observed mostly in beans fermented for 48 h.

After fermentation with *Saccharomyces cerevisiae* and *B. subtilis*, Dinh et al. [27] observed an increasing trend in time for the content of total polyphenols in green coffee beans; in fermented samples, the greatest total polyphenols content was acquired after 48 h. They suggested that complex polyphenols undergo depolymerization to simpler, more active forms during additional bioprocessing; however, prolonged processes might result in their loss due to oxidation. Similarly to the results obtained in this paper, the authors also noted significantly lower values for all fermented samples in comparison with those obtained for raw coffee material. Those observations might be a result of bean preparation, extraction or, as suggested by Dinh et al. [27], the consequence of chosen fermentation time. 

The content of 3-CQA is shown in Table 3. Both green and roasted fermented samples showed a trend similar to that described above for polyphenols—with longer fermentation duration, a significant increase in the amount of 3-CQA was observed. Green coffee samples fermented for 48 h exhibited higher values than their respective controls. After roasting, there was a great difference in 3-CQA content between control and fermented Arabica samples processed for durations less than 24 h with the difference diminishing with longer durations. In the case of Robusta beans, a notable difference between control and fermented roasted samples was evident after 6 h of processing. However, with longer durations, contents of 3-CQA further decreased in the controls, while in fermented samples, they increased. Unlike Arabica samples, Robusta beans fermented for 24 and 48 h exhibited higher amounts of 3-CQA than their controls. It shows that with a longer duration of bioprocessing positive changes in the amount of phenolic compounds extracted from green beans were more pronounced. Moreover, longer fermentation time positively affected the phenolic concentration in roasted beans.

**Figure 6:**
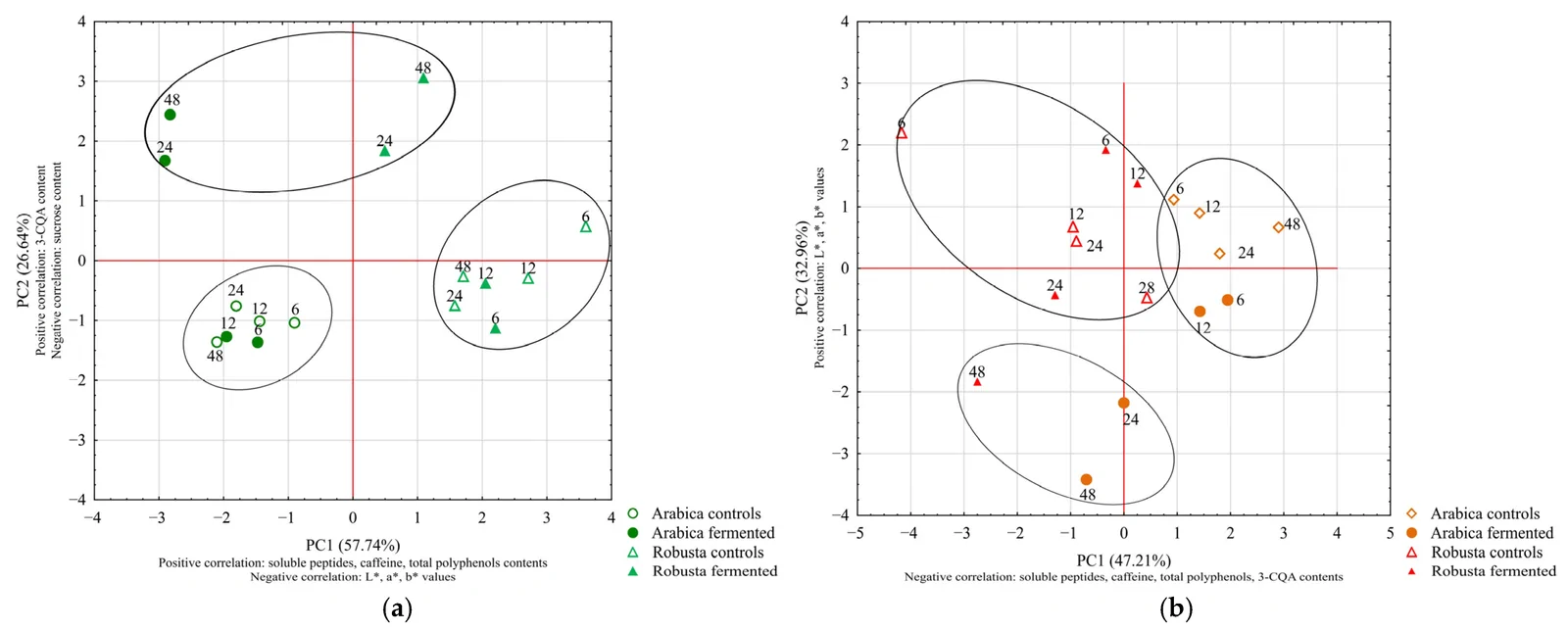
PCA score plots derived from color and composition data for green (**a**) and roasted (**b**) coffee beans fermented with *L. plantarum* ATCC 4008 with various durations, and their respective control samples (Experiment 3). Numbers above symbols indicate the time of fermentation [h]. Samples were divided into clusters (illustrated by the circles) based on cluster analysis.

The results of PCA analysis for green coffee are presented in Figure 6a. Significant changes in material were observed after more than 24 h of modification, which is confirmed by their clear distinction from other modified samples and controls. Figure 6b presents analysis performed for roasted coffee material. A similar trend regarding the influence of modification time can be observed; however, in the case of Robusta, only sample modified for 48 h was grouped separately from all other Robusta material. This was due to fundamental changes in sample composition and appearance caused by roasting itself.

## 4. Conclusions

Among the bacteria associated with the civet digestive tract, *B. subtilis* ATCC 6633, *Gluconobacter* sp. KKP 3751 and *L. plantarum* ATCC 4008 exhibited the best ability to grow in green coffee extract. These strains changed green coffee bean composition, mostly regarding precursors of color and aroma compounds (carbohydrates and soluble peptides). Those changes might have an interesting application for creating the quality of the final product. In the case of roasted beans, *L. plantarum* showed the potential to enhance the amount of antioxidants extracted from coffee.

The results showed that periods shorter than 24 h significantly reduced the amount of bioactive compounds available in coffee material, while with an extended duration, the positive effect on both green and roasted beans was pronounced. Those observations could be associated with microbial growth dynamics, i.e., obtaining the stationary growth phase. Further research regarding sensory quality and other factors (e.g., roasting level, brewing method) is needed to accurately determine the true potential of the tested bacterial strains to create unique coffee products.

## Figures and Tables

**Table 1:** Cell count (in log CFU × mL^−1^) at the onset and after 24 h of coffee fermentation with selected bacteria (Experiment 2).

Bacterium	Time [h]	
	0	24
	Arabica	
*B. subtilis* ATCC 6633	5.43 ± 0.26 ^a^	8.12 ± 0.16 ^a^
*Gluconobacter* sp. KKP 3751	6.87 ± 0.05 ^a^	8.29 ± 0.09 ^b^
*L. plantarum* ATCC 4008	6.01 ± 0.07 ^a^	8.56 ± 0.03 ^b^
	Robusta	
*B. subtilis* ATCC 6633	5.84 ± 0.03 ^a^	7.92 ± 0.42 ^a^
*Gluconobacter* sp. KKP 3751	6.82 ± 0.23 ^a^	8.37 ± 0.09 ^b^
*L. plantarum* ATCC 4008	5.86 ± 0.21 ^a^	8.75 ± 0.08 ^b^

The results presented are as average ± standard deviations (n = 2). For each bacterium, results with various lowercase letters are significantly different, as indicated by statistical analysis.

**Table 2:** Composition of coffee beans fermented with selected bacteria and their respective controls obtained in Experiment 2.

	Fructose [g/100 g dm]	Glucose [g/100 g dm]	Sucrose [g/100 g dm]	Soluble Peptides [g/100 g dm]	Caffeine [g/100 g dm]	Total Polyphenols [g/100 g dm]	3-CQA [g/100 g dm]
Green Arabica							
Control	0.13 ± 0.01 ^b^	0.12 ± 0.01 ^b^	3.28 ± 0.06 ^a^	10.31 ± 0.79 ^a^	0.66 ± 0.01 ^a^	1.54 ± 0.01 ^a^	0.89 ± 0.02 ^ab^
*B.subtilis* ATCC 6633	0.14 ± 0.03 ^b^	0.22 ± 0.04 ^a^	3.09 ± 0.05 ^b^	10.73 ± 0.46 ^a^	0.65 ± 0.01 ^a^	1.54 ± 0.02 ^a^	0.89 ± 0.01 ^a^
*Gluconobacter* sp. KKP 3751	0.25 ± 0.01 ^a^	0.16 ± 0.01 ^a^	2.67 ± 0.03 ^c^	9.96 ± 0.47 ^a^	0.64 ± 0.01 ^b^	1.39 ± 0.09 ^ab^	0.86 ± 0.01 ^b^
*L.plantarum* ATCC 4008	traces	traces	2.89 ± 0.20 ^abc^	11.21 ± 1.57 ^a^	0.63 ± 0.02 ^ab^	1.38 ± 0.01 ^b^	0.82 ± 0.04 ^ab^
Green Robusta							
Control	0.12 ± 0.01 ^b^	0.11 ± 0.01 ^c^	2.32 ± 0.04 ^a^	16.21 ± 0.38 ^ab^	1.03 ± 0.01 ^b^	1.90 ± 0.08 ^ab^	0.88 ± 0.01 ^b^
*B.subtilis* ATCC 6633	0.12 ± 0.02 ^b^	0.19 ± 0.01 ^a^	2.16 ± 0.18 ^ab^	17.60 ± 1.89 ^a^	1.05 ± 0.04 ^ab^	1.90 ± 0.18 ^ab^	0.91 ± 0.06 ^ab^
*Gluconobacter* sp. KKP 3751	0.21 ± 0.01 ^a^	0.13 ± 0.01 ^b^	2.01 ± 0.05 ^b^	15.23 ± 0.10 ^b^	1.06 ± 0.02 ^b^	1.91 ± 0.01 ^b^	0.88 ± 0.01 ^b^
*L.plantarum* ATCC 4008	0.07 ± 0.01 ^c^	traces	2.44 ± 0.03 ^a^	14.70 ± 0.77 ^b^	1.13 ± 0.02 ^a^	2.07 ± 0.02 ^a^	1.05 ± 0.02 ^a^
Roasted Arabica							
Control	n.d.	n.d.	n.d.	8.69 ± 0.22 ^b^	0.46 ± 0.03 ^ab^	1.00 ± 0.07 ^ab^	0.31 ± 0.02 ^a^
*B.subtilis* ATCC 6633	n.d.	n.d.	n.d.	8.82 ± 0.23 ^b^	0.46 ± 0.01 ^a^	1.08 ± 0.11 ^ab^	0.30 ± 0.01 ^a^
*Gluconobacter* sp. KKP 3751	n.d.	n.d.	n.d.	9.09 ± 0.71 ^ab^	0.44 ± 0.01 ^b^	0.94 ± 0.03 ^b^	0.29 ± 0.01 ^a^
*L.plantarum* ATCC 4008	n.d.	n.d.	n.d.	9.70 ± 0.63 ^a^	0.46 ± 0.03 ^ab^	1.04 ± 0.02 ^a^	0.30 ± 0.01 ^a^
Roasted Robusta							
Control	n.d.	n.d.	n.d.	11.86 ± 2.10 ^ab^	0.60 ± 0.02 ^b^	1.01 ± 0.03 ^a^	0.29 ± 0.01 ^b^
*B.subtilis* ATCC 6633	n.d.	n.d.	n.d.	14.03 ± 0.47 ^a^	0.72 ± 0.03 ^a^	1.29 ± 0.10 ^a^	0.34 ± 0.01 ^a^
*Gluconobacter* sp. KKP 3751	n.d.	n.d.	n.d.	13.04 ± 0.53 ^ab^	0.69 ± 0.02 ^a^	1.17 ± 0.10 ^a^	0.31 ± 0.02 ^ab^
*L.plantarum* ATCC 4008	n.d.	n.d.	n.d.	12.78 ± 0.62 ^b^	0.69 ± 0.06 ^ab^	1.25 ± 0.14 ^a^	0.34 ± 0.03 ^a^

The results presented are as average ± standard deviations (n ≥ 3). For each group of samples, superscript letters indicate homogenous groups created after analysis of variance. n.d.—not detected.

**Table 3:** Composition of control and *L. plantarum*-fermented coffee beans processed with various duration times (Experiment 3).

Time [h]		Fructose [g/100 g dm]	Glucose [g/100 g dm]	Sucrose [g/100 g dm]	Soluble Peptides [g/100 g dm]	Caffeine [g/100 g dm]	Total Polyphenols [g/100 g dm]	3-CQA [g/100 g dm]
Green Arabica								
6	C	0.18 ± 0.01 ^a^	0.17 ± 0.01 ^a^	3.38 ± 0.06 ^a^	14.14 ± 0.46 ^a^	0.77 ± 0.01 ^a^	1.48 ± 0.09 ^a^	1.12 ± 0.05 ^a^
	LP	0.15 ± 0.02 ^A^	0.15 ± 0.02 ^A^	3.57 ± 0.36 ^A^	12.49 ± 0.60 ^A^	0.78 ± 0.05 ^A^	1.57 ± 0.26 ^AB^	1.01 ± 0.10 ^AB^
12	C	0.15 ± 0.04 ^a^	0.14 ± 0.03 ^a^	3.11 ± 0.10 ^b^	13.94 ± 0.22 ^a^	0.72 ± 0.01 ^b^	1.39 ± 0.04 ^a^	1.04 ± 0.05 ^a^
	LP	0.08 ± 0.02 ^B^	0.08 ± 0.02 ^B^	3.10 ± 0.11 ^A^	12.60 ± 0.16 ^A^	0.73 ± 0.03 ^A^	1.42 ± 0.02 ^B^	0.93 ± 0.06 ^B^
24	C	0.14 ± 0.01 ^a^	0.15 ± 0.01 ^a^	2.70 ± 0.02 ^c^	11.98 ± 0.28 ^b^	0.68 ± 0.01 ^c^	1.34 ± 0.09 ^a^	1.02 ± 0.05 ^ab^
	LP	traces	traces	2.71 ± 0.01 ^B^	12.94 ± 0.04 ^A^	0.75 ± 0.01 ^A^	1.56 ± 0.01 ^A^	1.06 ± 0.03 ^AB^
48	C	0.13 ± 0.02 ^a^	0.13 ± 0.01 ^a^	2.49 ± 0.01 ^d^	11.26 ± 0.24 ^b^	0.62 ± 0.01 ^d^	1.29 ± 0.08 ^a^	0.91 ± 0.04 ^b^
	LP	n.d.	traces	2.24 ± 0.07 ^C^	12.21 ± 0.78 ^A^	0.72 ± 0.01 ^A^	1.58 ± 0.01 ^A^	1.13 ± 0.05 ^A^
Green Robusta								
6	C	0.13 ± 0.03 ^a^	0.13 ± 0.02 ^a^	2.33 ± 0.02 ^a^	24.42 ± 0.12 ^a^	1.10 ± 0.01 ^b^	2.31 ± 0.05 ^a^	1.26 ± 0.04 ^a^
	LP	0.14 ± 0.02 ^A^	0.13 ± 0.01 ^A^	2.29 ± 0.17 ^A^	20.97 ± 0.91 ^A^	1.11 ± 0.06 ^A^	1.93 ± 0.17 ^AB^	0.98 ± 0.07 ^B^
12	C	0.15 ± 0.01 ^a^	0.15 ± 0.01 ^a^	2.17 ± 0.02 ^b^	22.51 ± 0.19 ^b^	1.13 ± 0.01 ^a^	2.01 ± 0.03 ^b^	1.09 ± 0.04 ^b^
	LP	0.08 ± 0.01 ^B^	0.08 ± 0.02 ^B^	2.14 ± 0.03 ^A^	17.93 ± 0.71 ^B^	1.13 ± 0.01 ^A^	2.11 ± 0.09 ^AB^	1.02 ± 0.03 ^B^
24	C	0.12 ± 0.01 ^a^	0.12 ± 0.01 ^a^	1.99 ± 0.01 ^c^	17.52 ± 0.06 ^d^	1.06 ± 0.01 ^c^	1.80 ± 0.01 ^c^	1.01 ± 0.04 ^b^
	LP	0.05 ± 0.01 ^C^	0.05 ± 0.01 ^B^	1.81 ± 0.01 ^A^	19.46 ± 0.25 ^A^	1.12 ± 0.01 ^A^	1.94 ± 0.03 ^B^	1.04 ± 0.03 ^B^
48	C	0.15 ± 0.01 ^a^	0.16 ± 0.02 ^a^	1.98 ± 0.01 ^c^	18.25 ± 0.18 ^c^	1.06 ± 0.01 ^c^	1.93 ± 0.03 ^b^	1.05 ± 0.05 ^b^
	LP	traces	traces	1.44 ± 0.01 ^A^	17.67 ± 0.12 ^B^	1.15 ± 0.02 ^A^	2.14 ± 0.07 ^A^	1.21 ± 0.04 ^A^
Roasted Arabica								
6	C	n.d.	n.d.	n.d.	9.69 ± 0.04 ^b^	0.62 ± 0.01 ^a^	1.38 ± 0.03 ^a^	0.36 ± 0.01 ^a^
	LP	n.d.	n.d.	n.d.	10.28 ± 0.19 ^A^	0.53 ± 0.01 ^A^	1.06 ± 0.01 ^B^	0.25 ± 0.02 ^C^
12	C	n.d.	n.d.	n.d.	9.96 ± 0.03 ^a^	0.55 ± 0.01 ^b^	1.20 ± 0.01 ^b^	0.35 ± 0.01 ^a^
	LP	n.d.	n.d.	n.d.	10.78 ± 0.63 ^A^	0.55 ± 0.03 ^A^	1.13 ± 0.09 ^B^	0.25 ± 0.01 ^BC^
24	C	n.d.	n.d.	n.d.	9.16 ± 0.12 ^c^	0.55 ± 0.01 ^b^	1.24 ± 0.02 ^b^	0.31 ± 0.01 ^b^
	LP	n.d.	n.d.	n.d.	9.77 ± 1.17 ^A^	0.55 ± 0.02 ^A^	1.38 ± 0.07 ^A^	0.30 ± 0.01 ^A^
48	C	n.d.	n.d.	n.d.	9.30 ± 0.09 ^c^	0.46 ± 0.01 ^c^	0.96 ± 0.05 ^c^	0.29 ± 0.01 ^b^
	LP	n.d.	n.d.	n.d.	10.63 ± 0.34 ^A^	0.53 ± 0.03 ^A^	1.38 ± 0.12 ^AB^	0.28 ± 0.02 ^AB^
Roasted Robusta								
6	C	n.d.	n.d.	n.d.	14.57 ± 0.21 ^b^	1.01 ± 0.01 ^a^	2.18 ± 0.05 ^a^	0.59 ± 0.02 ^a^
	LP	n.d.	n.d.	n.d.	14.08 ± 0.89 ^A^	0.91 ± 0.02 ^AB^	1.50 ± 0.08 ^AB^	0.32 ± 0.04 ^AB^
12	C	n.d.	n.d.	n.d.	14.42 ± 0.11 ^b^	0.90 ± 0.01 ^b^	1.48 ± 0.06 ^b^	0.32 ± 0.01 ^b^
	LP	n.d.	n.d.	n.d.	13.25 ± 0.18 ^A^	0.86 ± 0.05 ^AB^	1.35 ± 0.06 ^B^	0.29 ± 0.01 ^B^
24	C	n.d.	n.d.	n.d.	16.60 ± 0.02 ^a^	0.77 ± 0.01 ^c^	1.39 ± 0.04 ^b^	0.29 ± 0.01 ^b^
	LP	n.d.	n.d.	n.d.	12.98 ± 1.02 ^A^	0.88 ± 0.01 ^B^	1.50 ± 0.01 ^B^	0.32 ± 0.02 ^AB^
48	C	n.d.	n.d.	n.d.	11.85 ± 0.07 ^c^	0.64 ± 0.01 ^d^	1.21 ± 0.02 ^c^	0.26 ± 0.01 ^c^
	LP	n.d.	n.d.	n.d.	13.10 ± 1.19 ^A^	0.92 ± 0.01 ^A^	1.73 ± 0.01 ^A^	0.34 ± 0.02 ^A^

The results are presented as average ± standard deviations (n ≥ 3). For each group of samples, superscript letters indicate homogenous groups created after analysis of variance: lowercase letters signify differences between controls; uppercase letters between fermented samples. C—control samples; coffee beans soaked in water; LP—samples fermented with *L. plantarum* ATCC 4008. n.d.—not detected.

## Data Availability

The data presented in this study are openly available on Zenodo at https://doi.org/10.5281/zenodo.13384388 (published on 30.08.2024).

## Supplementary Materials

The following supporting information can be downloaded at: https://www.mdpi.com/article/10.3390/foods14071143/s1, Table S1: Optical density changes (600 nm) after specified incubation times obtained in Experiment 1; Figure S1: Color parameters of green (1–3) and roasted (4–6) coffee beans prepared during Experiment 2; Figure S2: Color parameters of green (1–3) and roasted (4–6) Arabica coffee beans fermented with *L. plantarum* (LP) with various duration times, and their respective controls (Experiment 3); Figure S3: Color parameters of green (1–3) and roasted (4–6) Robusta coffee beans fermented with *L. plantarum* (LP) with various duration times, and their respective controls (Experiment 3).

## Abbreviations

The following abbreviations are used in this manuscript:

LAB	Lactic acid bacteria
CFU	Colony-forming bacteria
MRS	de Man, Rogosa and Sharpe medium
GY	Glucose and yeast medium
TBS	Tryptic soy broth
3-CQA	3-caffeoylquinic acid
OD	Optical density
HPLC	High-pressure liquid chromatography
ELSD	Evaporative light-scattering detector
PDA	Photodiode detector
ANOVA	One-way analysis of variance
PCA	Principal component analysis
dm	Dry matter
n.d.	Not detected
