# Supplementary material for: Composition of Coffee Beans Influenced by Bioprocessing with Selected Bacteria
Source: Foods. 2025 Mar 25;14(7):1143. doi: 10.3390/foods14071143 (PMC11988858; doi:10.3390/foods14071143)
Supplement: Supplementary file 1 [file foods-14-01143-s001.zip › foods-3530729-supplementary.pdf]

Table S1: Optical density changes (600 nm) after specified incubation times obtained in Experiment 1.

Time [h]		***B.subtilis* ATCC 6633**	***Gluconobacter* sp. KKP 3751**	***L.plantarum* ATCC 4080**	***L.brevis* DSMZ 20053**	***L.brevis* from Biolacta, Poland**
**24**	CM	0.37±0.05 ^{c}	0.61±0.06 ^{b}	0.71±0.12 ^{ab}	0.56±0.09 ^{b}	0.82±0.02 ^{a}
	CE	0.45±0.03 ^{a}	0.49±0.01 ^{a}	0.47±0.06 ^{a}	0.29±0.04 ^{b}	0.44±0.04 ^{a}
	rel.	123.8%	80.0%	65.7%	51.2%	52.9%
**48**	CM	0.46±0.19 ^{b}	0.84±0.04 ^{a}	1.01±0.11 ^{a}	0.98±0.01 ^{a}	0.91±0.05 ^{a}
	CE	0.74±0.03 ^{a}	0.81±0.03 ^{a}	0.70±0.05 ^{ab}	0.53±0.09 ^{ab}	0.65±0.02 ^{b}
	rel.	161.8%	97.3%	68.9%	54.1%	70.8%

Results presented as average ± standard deviations (n = 4). Changes calculated by subtracting values obtained at the beginning of the screening (0 h) from values measured after specified incubation periods. In each row, superscript letters indicate homogenous groups created after analysis of variance. CM – control medium; tryptic soy broth, glucose and yeast extract medium, de Man, Rogosa and Sharpe medium for *B.subtilis*, *Gluconobacter* sp. and LAB, respectively; CE – green coffee extract; rel. - relative change in mean values between coffee extract and control medium.

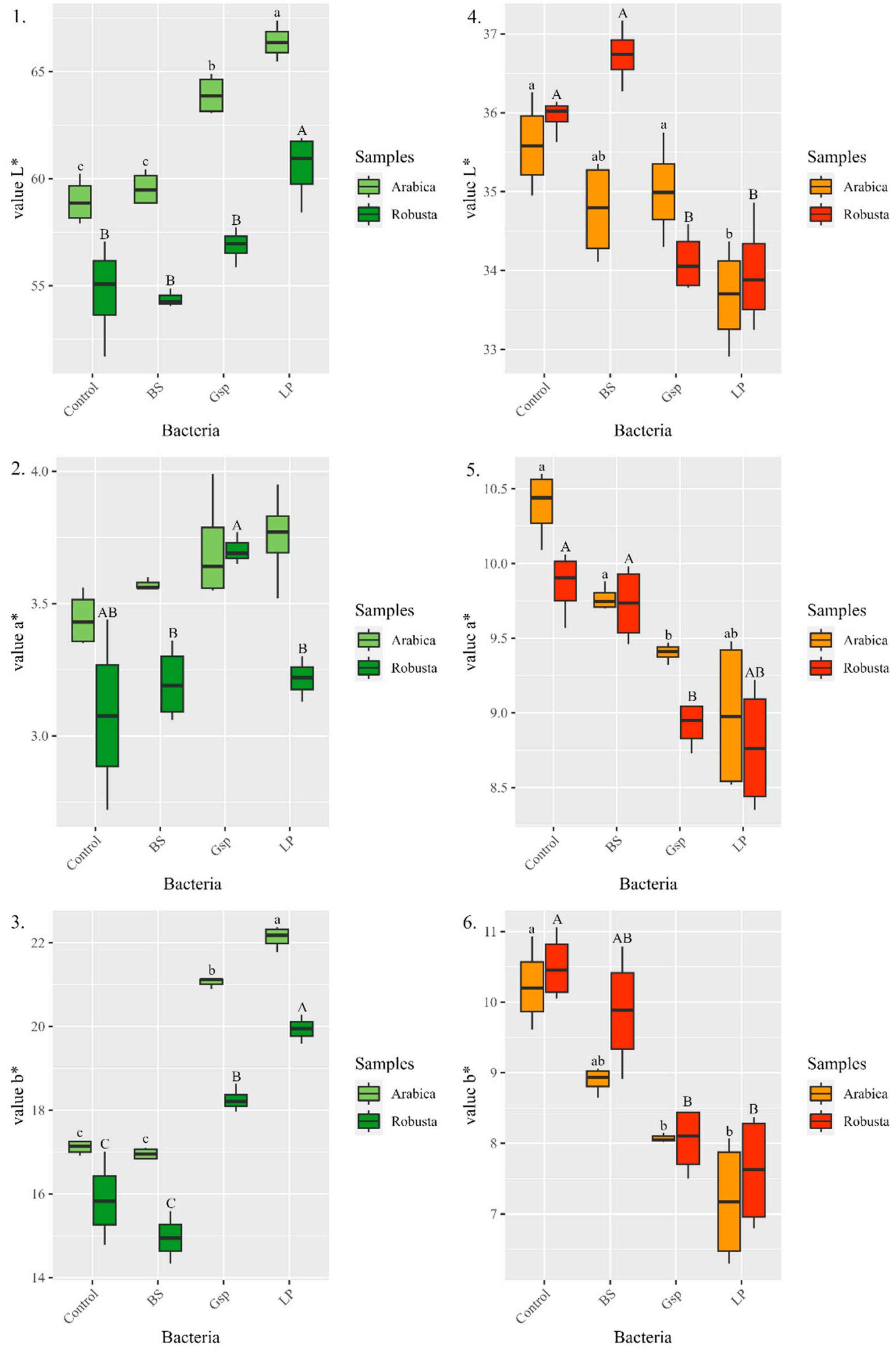

Figure S1: Color parameters of green (1-3) and roasted (4-6) coffee beans prepared during Experiment 2.

BS, Gsp, LP indicate samples fermented with *B. subtilis* ATCC 6633, *Gluconobacter* sp. KKP 3751 and *L. plantarum* ATCC 4080, respectively. Letters indicate homogenous groups created after analysis of variance, lowercase letters signify differences between Arabica samples, uppercase letters between Robusta samples. Results without letter indicators did not differ significantly.

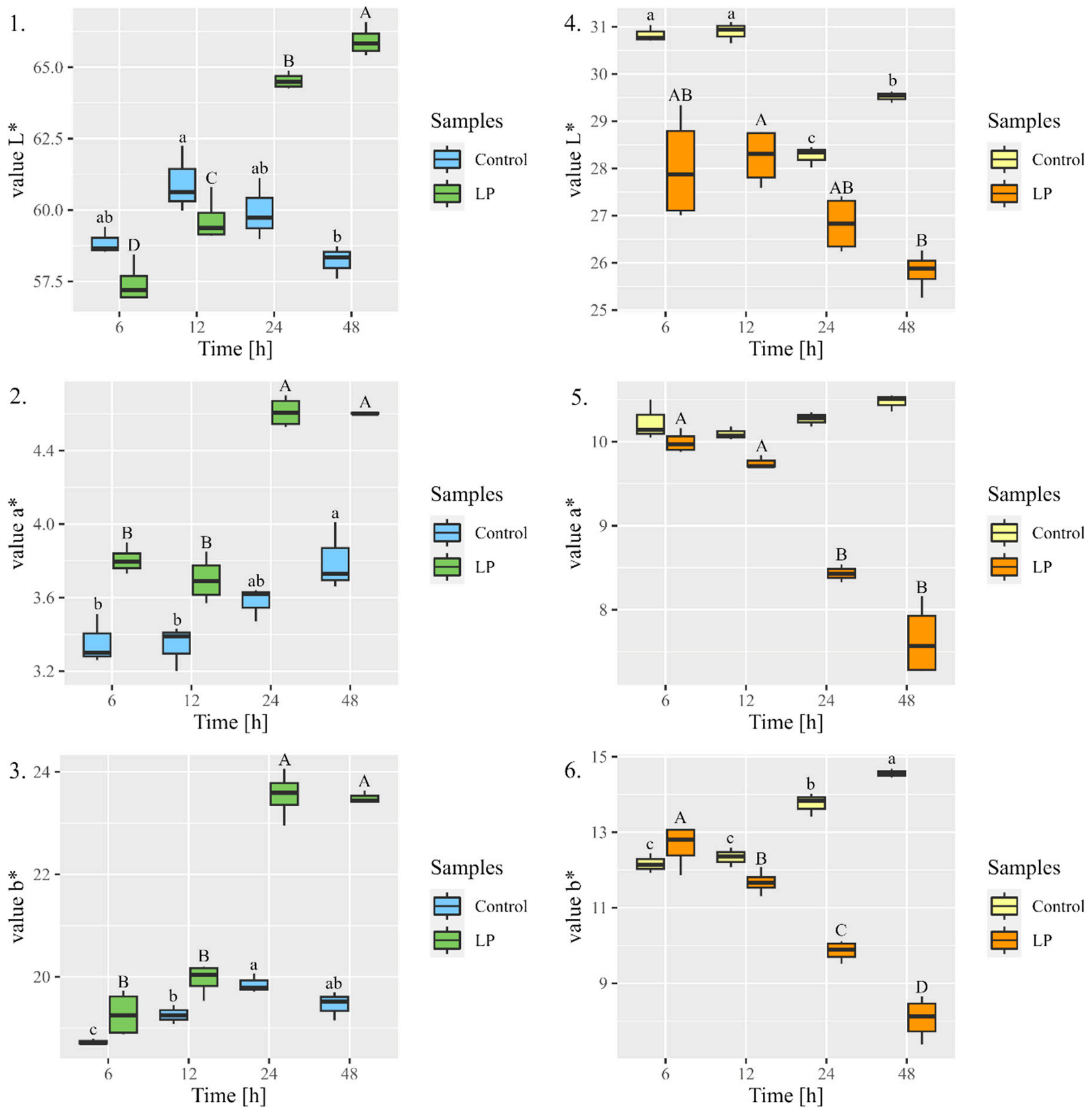

Figure S2: Color parameters of green (1-3) and roasted (4-6) Arabica coffee beans fermented with *L. plantarum* (LP) with various duration times and their respective controls (Experiment 3).

Letters indicate homogenous groups created after analysis of variance, lowercase letters signify differences between controls, uppercase letters between fermented samples. Results without letter indicators did not differ significantly.

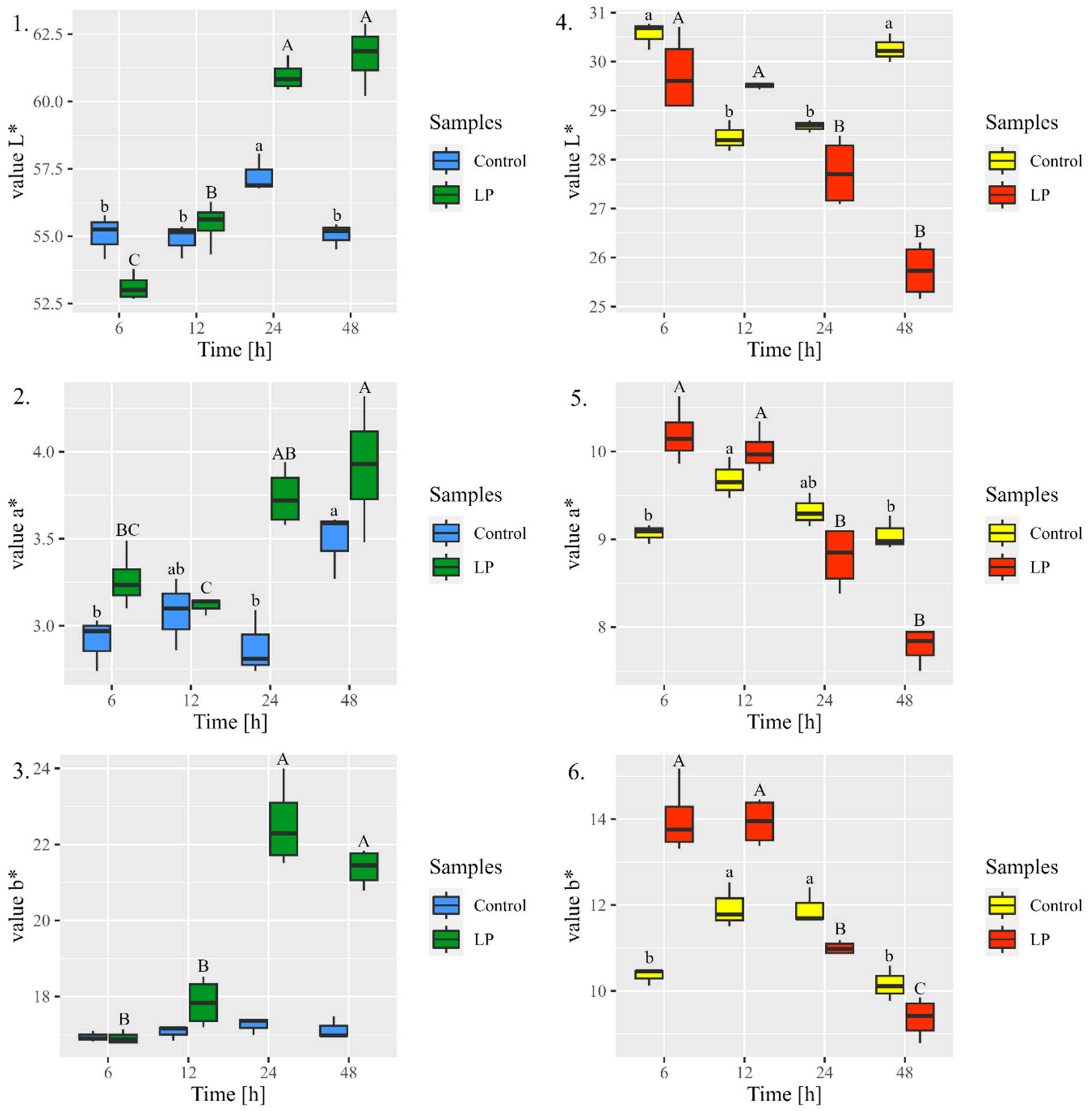

Figure S3: Color parameters of green (1-3) and roasted (4-6) Robusta coffee beans fermented with *L. plantarum* (LP) with various duration times and their respective controls (Experiment 3).

Letters indicate homogenous groups created after analysis of variance, lowercase letters signify differences between controls, uppercase letters between fermented samples. Results without letter indicators did not differ significantly.
